# New Evaluation Procedure for Multi-Dimensional Mechanical Strains and Tangent Moduli of Breast Implants: IDEAL IMPLANT^®^ Structured Breast Implant Compared to Silicone Gel Implants

**DOI:** 10.3390/bioengineering6020043

**Published:** 2019-05-12

**Authors:** Harold J. Brandon, Larry S. Nichter, Dwight D. Back

**Affiliations:** 1 Department of Mechanical Engineering and Materials Science, Washington University, St. Louis, MO 63130, USA; 2 Pacific Center for Plastic Surgery, Newport Beach, CA 92660, USA; lnichter@imagedr.com; 3 Department of Plastic Surgery, University of California Irvine, Irvine, CA 92868, USA; 4 Medical Device Pros, LLC, Satellite Beach, FL 32937, USA; dback@MedicalDeviceProsLLC.com

**Keywords:** dual lumen, breast implant, breast implant testing, structured, tangent modulus, IDEAL IMPLANT, strain, silicone gel, breast implant mechanical properties, compression, breast implant rupture, breast implant evaluation, capsular contracture, structured saline breast implant, breast implant dimensions

## Abstract

The IDEAL IMPLANT^®^ Structured Breast Implant is a dual lumen saline-filled implant with capsular contracture and deflation/rupture rates much lower than single-lumen silicone gel-filled implants. To better understand the implant’s mechanical properties and to provide a potential explanation for these eight-year clinical results, a novel approach to compressive load testing was employed. Multi-dimensional strains and tangent moduli, metrics describing the shape stability of the total implant, were derived from the experimental load and platen spacing data. The IDEAL IMPLANT was found to have projection, diametric, and areal strains that were generally less than silicone gel implants, and tangent moduli that were generally greater than silicone gel implants. Despite having a relatively inviscid saline fill, the IDEAL IMPLANT was found to be more shape stable compared to gel implants, which implies potentially less interaction with the capsule wall when the implant is subjected to compressive loads. Under compressive loads, the shape stability of a higher cross-link density, cohesive gel implant was unexpectedly found to be similar to or the same as a gel implant. In localized diametric compression testing, the IDEAL IMPLANT was found to have a palpability similar to a gel implant, but softer than a cohesive gel implant.

## 1. Introduction

Saline-filled and silicone gel-filled breast implants that are marketed today have undergone extensive mechanical testing in order to demonstrate their strength and durability. Breast implant manufacturers have followed the recommendations provided in the FDA guidance document for pre-market approval (PMA) applications [1] which requires specific types of mechanical testing and expected strength and durability requirements of silicone shells and the finished product. Many of the current mechanical evaluations utilize ASTM and ISO standards [2,3]. Mechanical testing that replicates clinical conditions is the goal of these experimental procedures, but this is difficult to achieve in the laboratory. Instead, breast implant manufacturers have used conventional experimental techniques that can be performed in any well-equipped mechanical testing laboratory. The tests used to demonstrate implant durability are rigorous and repeatable. Current routine implant testing includes tensile strength, ultimate elongation, tear resistance, joint testing, cyclic fatigue testing, ultimate strength tests, and valve competency (for saline implants). Although the mechanical tests have not exactly simulated the in vivo environment, the resulting data have significantly aided in the development and design of modern-day breast implants. Breast implants being implanted today have increased strength characteristics and have proven to be durable in vivo.

There are several other advantages to testing using conventional experimental techniques. Testing under standardized, repeatable conditions yields baseline data that allow different designs to be compared using the same experimental technique. Another important aspect of tests conducted under standardized, repeatable conditions is that they provide data from explanted implants which can be used to evaluate the effects of long-term implantation on the strength and durability of breast implants. This has been accomplished by mechanically testing retrieved explant shells in the same manner as the control shells and comparing the explant and control data [4,5]. Explant performance has also been evaluated by comparing strength data with ASTM standards [6]. The implant’s multi-dimensional strain and the change in associated implant shape due to compression is a mechanical property that has not been previously evaluated for breast implants. Implant strain is directly related to implant shape change.

A test methodology that simulates in vivo conditions would be the desired procedure for this study, but this is difficult to achieve in the laboratory. The experimental approach taken in this study is to use a conventional experimental set up that has proven useful in verifying implant durability during compression, and at the same time is amenable to a theoretical analysis. The methodology used in this study is compressive loading of an implant between two parallel plates. Compression testing between two parallel plates with air or saline has been used previously by implant manufacturers as described in their PMA summary of safety and effectiveness data [7,8,9,10,11], and by other implant researchers [12] for cyclic fatigue and ultimate strength testing. Flat plate compression testing has been used to provide implant cyclic fatigue failure characteristics, data for fatigue lifetime predictions, ultimate strength, and morphological features of fatigue failure. All implants tested in the study are the finished commercial product that were not previously implanted. Explants are not considered in this study.

In this study we describe an experimental and theoretical analysis of the geometric, or shape change, response of three different types of smooth surface breast implants to compressive loading. Jewell et al. [13] measured the form stability, or shape retention, of gel implants as an implant moves between a horizontal and vertical orientation. These studies did not characterize the shape retention of implants when subjected to compressive loading. Resistance to gel deformation was also measured [13] using biomechanical tissue characterization (BTC), however, these studies characterized the gel fill rather than the total implant deformation and shape change. Although there is an abundance of published mechanical property data for breast implants, no published study could be found in the literature on the overall, or total, implant strain and associated shape change due to compressive loading.

**Figure 1:**
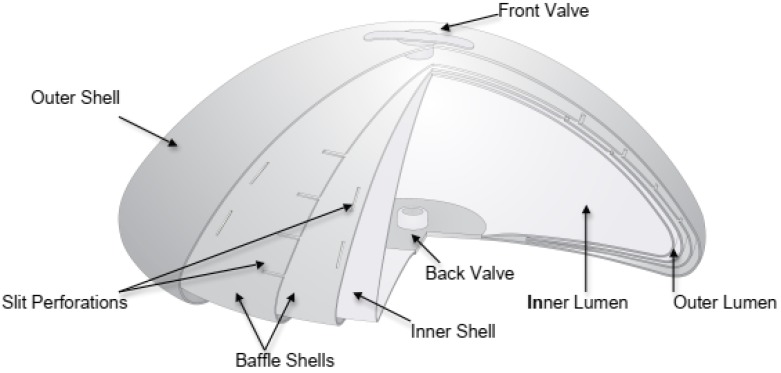
Cut-away Drawing of the IDEAL IMPLANT.

A cut-away drawing of a representative IDEAL IMPLANT (335 cc to 555 cc size) is provided in Figure 1 detailing its structure. The IDEAL IMPLANT is a smooth surface implant comprised of two lumens within a series of nested shells made from cross-linked silicone (polydimethylsiloxane) elastomer that are attached at the patch on the back of the implant. The inner lumen within the inner shell is filled through a valve in the posterior patch. The outer lumen within the outer shell and surrounding the inner shell is filled through a valve on the anterior surface. Unattached and floating within the outer lumen is a baffle structure designed to control movement of the saline in the outer lumen. The resistance to saline movement and additional structure of the implant has been described by some plastic surgeons as giving the implant an “apparent” viscosity that is similar to the viscosity of silicone gel. This baffle structure is comprised of one to three nested baffle shells that are perforated with slits so the saline is free to move through the slits, as well as around and between the baffle shells. In addition, the internal structure supports the outer shell so the upper portion of the implant does not collapse when the patient is upright and reduces the ability of the outer shell to fold upon itself and crease.

Although the structured IDEAL IMPLANT is saline-filled, it is a distinctly different type of implant than unstructured single-lumen saline-filled implants or silicone gel-filled implants, making it a third type of breast implant [14].

Table 1 and Table 2 summarize recent clinical trial results for deflation/rupture and capsular contracture (CC) rates for the IDEAL IMPLANT and the three silicone gel implants that have been approved by the FDA for marketing in the US over 7–8 years [15,16,17,18]. Per Table 1, the IDEAL IMPLANT has a rupture rate of 2.1% compared to a range of 7.4% to 13.6% for the gel implants. Per Table 2, the CC rate for the IDEAL IMPLANT is 6.6% compared to a range of 10.9% to 16.2% for the gel implants. The scar tissue that normally surrounds a breast implant is called a “capsule”. If this scar tissue contracts, called “capsular contracture”, the implant is compressed and feels firmer when palpated. The Baker scale, Grades I through IV, is used to quantitate CC [19] as follows:

Grade I: Breast normally soft and looks natural
Grade II: Breast slightly firm but looks normal
Grade III: Breast firm with visible distortion
Grade IV: Breast hard, painful, and greater distortion

The silicone gel implants listed in Table 1 and Table 2 have mechanical properties that have been approved by the FDA during the respective PMA applications. These properties include shell tensile strength, shell ultimate elongation, shell thickness, as well as cyclic fatigue and ultimate strength characteristics. Interestingly, and perhaps related to the favorable clinical data, the ultimate strength of the IDEAL IMPLANT exceeds 15.2 kN, and is believed to exceed the ultimate strengths of all saline and gel-filled implants available commercially. There also have been no fold flaw failures of the IDEAL IMPLANT in the clinical trial through eight years. Fold flaws are a cause of implant failure when the shell of an implant is flexed or folded repeatedly, weakening the shell along the fold line and eventually leading to a perforation [20]. A more stable implant geometry, perhaps due to the internal structure, may be a factor in eliminating or reducing fold flaw failures.

Given the substantially lower CC rates, lower deflation/rupture rates, especially the absence of fold flaw failures, and the high ultimate strength of the IDEAL IMPLANT, a primary motivation for this study was to investigate characteristics of the IDEAL IMPLANT that might be substantially different when compared to current single-lumen implants. Since the individual shells used in the IDEAL IMPLANT have a thickness that is substantially the same as other breast implants, this does not seem to be a distinguishing feature that could explain the lower deflation/rupture and CC rates.

With respect to implant deflation/rupture, Brandon et al. [12] observed two different modes of implant failure which depended on the magnitude of the cyclic load and frequency. A tear mode occurred at high loads and few cycles to failure, while a pinhole mode occurred at low loads and many cycles to failure. Both modes of failure occurred in the shell outer perimeter. However, no research could be identified on the effect of, or relationship between, the types of implant movement within the breast capsule that might affect CC. In particular, it is unclear whether CC is influenced by low loads at high frequency, high loads at a low frequency, very high loads at occasional frequency, or a combination thereof.

Malahias et al. [21] provide a literature review on CC, including a summary of theories on the etiology of CC. One leading theory for the pathogenesis of CC involves the alignment and contraction of fibroblasts within the scar capsule that forms around a breast implant [22]. By this theory, the risk of CC increases with smooth surface implants because capsule fibroblasts can align to the implant surface in a planar structure, whereas a textured implant surface disrupts the fibroblast planar structure. For silicone gel implants, the CC rates in Table 2 include both smooth and textured surface silicone gel implants, so the CC rates could be higher if only smooth surface gel implants were studied. Given that the IDEAL IMPLANT is a smooth surface implant, this makes the lower CC rates observed for the IDEAL IMPLANT more striking.

Derby and Codner [23] provide a review of core clinical data for textured silicone breast implants. As part of this review, the concepts of implant counter-pressure and tissue surface friction are used to hypothesize physical mechanisms that could help explain observed differences in CC between textured and smooth implants, and between highly cohesive gel and earlier generation implants. Both of these concepts can be related to the movement of the implant, or its surface, relative to the breast capsule, or in an even broader sense, the shape stability of an implant.

Other theories for CC can also be related to the interaction of the implant with the capsule wall. For instance, it is known that repeated small amounts of trauma to tissue can stimulate inflammation and scarring. Because an implant’s response to a load can result in varying amounts of implant-capsule wall interaction through friction and stresses, understanding an implant’s response to loads may be crucial in understanding CC and other untoward outcomes involving the capsule, such as breast implant-associated anaplastic large cell lymphoma (BIA-ALCL), double capsule formation, seroma formation and associated breast implant illness (BII). In particular, the relative movement of an implant when subjected to a compressive load, i.e., the overall strain that an implant experiences during compression loading, may be relevant to the degree of interaction that an implant has with the surrounding breast capsule in vivo. This interaction, caused by repeated compressive loading on an implant, may be the repeated “micro” trauma to the capsule that stimulates CC and other chronic inflammatory conditions involving the capsule, and that may be related to the development of BII or BIA-ALCL. Therefore, a more shape-stable implant could have less interaction with the surrounding breast capsule during repeated compressive loads, resulting in less repeated trauma to the capsule.

Many of the theories proposed to help explain the differences between implant types and observed clinical results relate to implant mechanical properties and the interface between the implant and breast capsule. This study describes and validates a methodology for characterizing implant mechanical properties related to implant mobility, or shape stability, which also has a relationship to ultimate strength and other total implant dynamics such as fold flaws and movement within the capsule when subjected to compressive loading.

## 2. Materials and Methods

**Figure 2:**
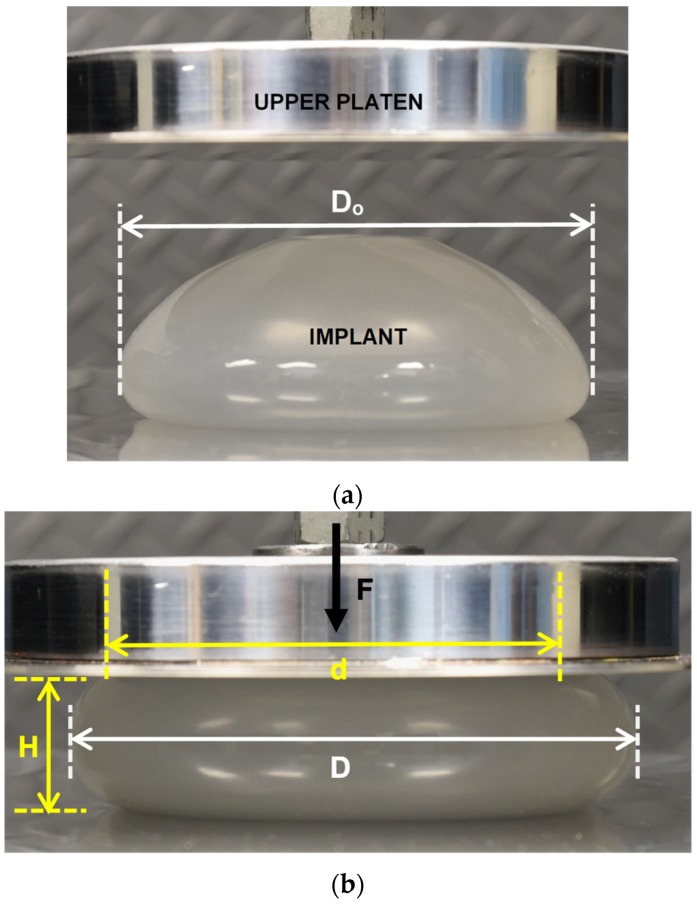
(**a**) IDEAL IMPLANT between Platens Prior to Compression Testing. (**b**) IDEAL IMPLANT Undergoing Compression (44.5 N) with Labelled Geometric Properties.

Unimplanted commercial implants were compressed between two parallel plates (platens) and data were analyzed using a new methodology. The laboratory apparatus used for testing was an Instron 5500R (Instron^®^, Norwood, MA, USA) equipped with a 1 kN load cell. This machine is computer-controlled and equipped with an automated data acquisition system. The Instron system measures the applied load versus the displacement. Data for the entire load–deflection curve is recorded by the computer system. The Instron can be operated with a variety of cross-head speeds producing a desired strain rate in the sample being tested. Both the upper and lower polished stainless-steel plates were very smooth, and had a measured surface roughness of about 0.18 micrometers. Figure 2a,b show the experimental setup for the compression testing. Figure 2a is a photograph of an implant prior to commencing the compression, and Figure 2b is a photograph of the same implant during a compression test at a load of 44.5 N.

For the compression experiments, each implant was loaded along a projection line from the center of the anterior (“front”) to the center of the posterior (“back”) of the implant. Compressive loading in this direction creates a maximum tensile stress around the perimeter, or equator, of the implant compared to much lower stresses on the anterior and the posterior regions of the implant. The primary variables for this study included the applied force (F), the implant projection between the platens (H), and the diameter of the implant (D) as illustrated in Figure 2b. The implant contact diameter (d) for the upper and lower platens can also be calculated directly by this methodology, but d is not required for the analysis described in this paper.

The load F and implant projection H, or plate spacing, were measured for all implants tested. The implant diameter D was then either measured or calculated as discussed in the next section. All other variables considered in this study were determined from these three primary variables, D, H and F. Implant internal pressures and shell stresses are not considered in this study, but will be presented in a future publication.

Three different types of implants were tested and analyzed in this study: an IDEAL IMPLANT, an Allergan Natrelle INSPIRA^®^ (Irvine, CA, USA) cohesive gel implant, and an Allergan Natrelle INSPIRA^®^ gel implant. The implant sizes represented a mid-range implant size, and the three implants were chosen so that their masses, volumes, initial diameters, and initial projections were as similar as possible. All three types of implants are approved by the FDA, and had previously undergone extensive mechanical testing for the PMA application [7,11]. Both Allergan gel implants were received in sealed sterilized packages. The IDEAL IMPLANT used in the testing was provided pre-filled with 0.9% USP sterile saline to the manufacturer’s recommended volumes. All three implants were round with smooth shells and were the finished sterilized product. Table 3 compares the geometric as well as other characteristics of the tested implants. The initial diameter D_o_ and initial plate spacing H_o_ were measured at a minimal load of 0.445 N except as noted for the Allergan cohesive gel implant. The implants are remarkably similar in size and dimensions.

Each implant was tested separately using two different test techniques: a static test and a dynamic test. All tests were conducted in air at a room temperature of 22 °C. The basic objective of the static test was to measure the implant diameter D as a function of the load F and the plate spacing H. In the static tests, each implant was first placed on the lower platen and the upper platen was lowered to generate an initial load of 0.445 N, and the initial implant diameter D_o_ was manually measured using calipers. The load F and the plate spacing H were recorded by the computer system. The implant was then compressed by manually dialing the position of the upper platen for a projection H to produce a load F of 22.2 N on the implant. Then F, D, and H were recorded as before. This process was continued for loads of 44.5, 66.7, 89.0, 133, 178, 222, 267, 356, 444, and 534 N. Approximately 30 to 60 seconds was needed to dial a load F and measure the diameter D. The contact diameter d of the implant with the platens was not considered for the static tests since it was not required for the analyses. Higher loads than typically experienced in vivo were considered because the results have application to cyclic fatigue and implant ultimate strength.

Following the static tests, each implant was tested dynamically by continuously applying an increasing load F to the implant at a constant rate. The initial step for dynamic testing consisted of placing the unloaded implant on the lower platen and manually dialing the position of the upper platen to produce a load of 0.445 N on the implant. The Instron system was then operated to continuously apply a load from 0.445 N to 534 N at a cross-head speed (strain rate) of 25.4 cm/minute. The load F and implant projection H were continuously measured by the computer system.

After the completion of the static and dynamic tests, the upper and lower plates were lubricated with a thin layer of silicone oil (DPDM 400) applied with a cloth. The dynamic tests were repeated with lubricated platens for all three implants using the identical test procedure used for the dry platen tests. The objective of the lubricated platen tests was to determine if implant-compressive load surface friction had an effect on implant shape change. During the lubrication tests, F and H were continuously recorded by the Instron computer system. As with the dry platen testing, the initial load on the implant was 0.445 N, the final load was 534 N, and the cross-head speed was 25.4 cm/minute.

Each implant was inspected during the course of the compression experiments, and no shell wear or damage was noted on any of the implant shells. After all the dry and lubricated platen tests were complete, the average shell thickness near the perimeter of the implants was measured using the following procedure. First, the implant was cut around the perimeter and the anterior side of the implant was removed for measurement. Second, the gel from the cohesive gel and gel implants was removed from the shells using a cloth and isopropyl alcohol. Third, the shell thickness was measured at four, equally spaced locations around the outer anterior perimeter using a caliper. The four measurements were then averaged to determine the average shell thickness (t). These average shell thicknesses for the three implants are included in Table 3. The cohesive gel implant shell is about 20% thicker than the IDEAL IMPLANT shell, and the gel implant shell is about 8.5% thicker than the IDEAL IMPLANT shell around the anterior outer perimeter.

The clinical relevance of this testing relates to expected high cyclic, low cyclic and occasional loads that a breast implant may be subjected to over its lifetime. Table 4 summarizes these loads that a breast or implant may be subjected to over its lifetime. Typical higher frequency cyclic loads that have been reported range from about 2 N to 60 N [24] for kneeling, walking, running, and stair-climbing, whereas very low frequency loads, such as lying down on a breast when sleeping or hugging, can generate forces of about 156 N or higher, depending on the weight of the individual and fraction of the body weight applied to the breast [25]. A CPR procedure could generate moderate frequency, higher loads up to about 556 N [26] over a short period of time. Non-cyclic forces exerted on a breast during a mammography [27] can range from about 49–187 N. Although these are typical loads that a breast might be subjected to, other combinations of activities (e.g., running up or down stairs, vigorous aerobic exercise) could generate cyclic or quasi-cyclic loads between these ranges. Consequently, the load test range of up to about 500 N was chosen for this study.

Lastly, a simple experimental study was conducted to better understand the mechanical response of an implant to a localized lateral compressive force across the implant diameter, such as pinching or grasping the implant at the perimeter. This differs from the total implant response to compressive loads, since the implant is not confined by platens and is able to move and respond freely to the lateral compressive load. This testing was meant to provide additional quantitative information about the strain response when an implant is diametrically pinched at the perimeter by finger tips at low loads. This type of localized strain entails relatively low forces of about 8 N or less and is related to implant tactility or palpability. These tests were conducted using a Torbal FC10 (Scientific Industries, Inc., Bohemia, NY, USA) 10 N push-pull digital force gauge configured with a simulated silicone finger.

## 3. Results

The first segment of the implant compression study consisted of (a) developing a geometric model for an implant undergoing compression between two platens, (b) validating the geometric model through static, manual implant geometry measurements compared to calculated implant geometry, and (c) validating a quasi-equilibrium assumption for the geometric model by comparing static compression measurements to dynamic compression calculations per the model. The purpose of developing and validating this approach was to provide a full geometric description of an implant undergoing compression using only the experimental plate spacing H and force F measurements that can be continuously monitored and recorded digitally during the compression of an implant in a load frame. The load F and plate spacing H measurements follow the same experimental procedure as that used currently for implant fatigue and ultimate strength testing. One implant size each for the IDEAL IMPLANT, Allergan Natrelle INSPIRA cohesive gel and Allergan Natrelle INSPIRA gel implant product lines were tested for this initial demonstration of a new method for evaluating total implant mechanical properties. The implant volumes and dimensions were carefully selected from within these product lines to eliminate differences in geometric factors from the analyses as much as possible (see Table 3).

The second segment of the study then utilized the validated geometric model and quasi-equilibrium assumption for a compressed implant to derive bulk, or total, implant mechanical properties. These total implant mechanical properties include compressive multi-dimensional strains and associated tangent moduli. These properties are mathematical descriptions for the shape stability of the implants.

Lastly, the effects of frictional forces between the implant and compressing platens were investigated by using both dry and lubricated platens. This provides for a more comprehensive understanding of how a breast implant may move in a spectrum of environments within the breast capsule and within the surrounding synovial fluid. Since an implanted breast implant may not be confined or attached to the wall of a breast capsule, and its surface may be “lubricated” to varying degrees, it is important to consider how the compressive and geometric properties are affected by lubrication as well as a “dry” surface.

### 3.1. Compressed Implant Geometry Model

As depicted in Figure 2b, an implant under compressive load can be described by the geometric factors d, D and H. A compressed implant is assumed to have a shape comprised of a central flattened cylinder, or “puck”, combined with the outer half of a torus. For a particular implant, the volume (V) is a constant during compression loading since the density of the fill material is assumed constant and incompressible. The volume V of this assumed geometry is given by:

(1) $$\begin{array}{c} V=\left(\mathrm{volume}\;\mathrm{of}\;\mathrm{cylinder}\right)+\left(\mathrm{volume}\;\mathrm{of}\;\mathrm{outer}\;\mathrm{half}\;\mathrm{of}\;\mathrm{torus}\right),\\ V=\frac{{\pi d}^{2}H}{4}+2\pi \left(\frac{2H}{3\pi }+\frac{d}{2}\right)\frac{{\pi H}^{2}}{8} \end{array}$$

Equation (1) is a quadratic equation in d, having a positive root:

(2) $$d=\frac{1}{2}\left(-\frac{\pi H}{2}+\sqrt{\frac{16}{\pi }\frac{V}{H}+\left(\frac{\pi^{2}}{4}-\frac{8}{3}\right)H^{2}}\right)$$

Therefore, at each experimentally measured load F and plate spacing H, the value of d can be calculated. The implant diameter D at each load F can also be calculated at each experimental F and H as:

(3) D=H+d

Equation (3) assumes that the shape of the implant perimeter between the two platens is semi-circular having a diameter equal to H. After substituting Equation (2) into Equation (3), the calculated value of D becomes a function of two known variables, the measured implant projection or plate spacing H and the implant volume V:

(4) $$D=H+\frac{1}{2}\left(-\frac{\pi }{2}H+\sqrt{\frac{16}{\pi }\frac{V}{H}+\left(\frac{\pi^{2}}{4}-\frac{8}{3}\right)H^{2}}\right)$$

The implant diameter D and projection H during compression can now be used to calculate the surface area (A) of the implant, again assuming that the implant geometry is a composite of a cylinder and outer half of a torus:

(5) $$\begin{array}{c} A=\left(\mathrm{area}\;\mathrm{of}\;\mathrm{cylinder}\;\mathrm{faces}\right)+\left(\mathrm{area}\;\mathrm{of}\;\mathrm{outer}\;\mathrm{half}\;\mathrm{of}\;\mathrm{torus}\right),\\ A=\frac{{\pi d}^{2}}{2}+\frac{\pi^{2}\mathrm{dH}}{2}+{\pi H}^{2},\\ A=\frac{\pi }{2}D^{2}+\left(\frac{\pi^{2}}{2}+\pi \right)\mathrm{DH}+\left(\frac{3\pi }{2}-\frac{\pi^{2}}{2}\right)H^{2} \end{array}$$

By substituting Equation (4) for D into Equation (5), the implant surface area A becomes solely a function of measured H, which is a function of the measured load F profile during the dynamic testing and the known implant volume V.

Since a breast implant does not have the assumed flattened cylinder-outer torus composite geometry under no load and very low loads, the geometric implant model is considered to be the least accurate at very low compression loads.

### 3.2. Validation of Compressed Implant Geometric Model

To validate the compressed implant geometric model, static experimental measurements for the implant diameter D as a function of load F were compared to the calculated implant diameter D from the same static experiments using the experimentally measured implant projection H in Equation (4). For the static tests, an implant was compressed in steps, allowing for experimental measurements of the implant diameter D at each step as a function of F and H.

**Figure 3:**
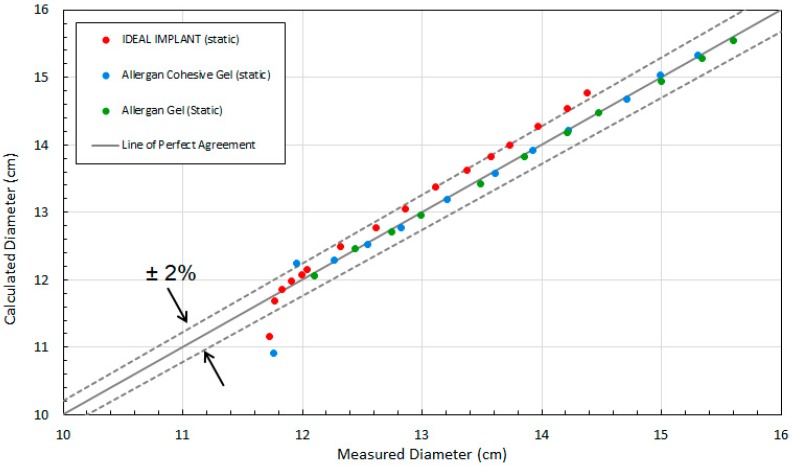
Comparison of Measured and Calculated Diameter (D) for Static Compression Testing.

Figure 3 shows the calculated implant diameter D compared to the measured implant diameter for the static compression testing. As shown, the implant diameters calculated using Equation (4) are within about ±2% of the measured implant diameters. The average error of the calculated diameters relative to the measured diameters for loads of 22.2 N to 534 N were found to be 1.7%, 0.17%, and −0.25% for the IDEAL IMPLANT, Allergan cohesive gel, and Allergan gel, respectively. The errors in calculated diameter D for the IDEAL IMPLANT are greater than those for the Allergan gel implants, although the agreement is still considered to be very good. As illustrated by Figure 3, the geometric model given by Equation (4) overestimates the implant diameter for the IDEAL IMPLANT, and this difference is believed to be related to the dual lumen structure of the implant which is not entirely accounted for by the Equation (4) geometric model. The overestimate for the diameter using Equation (4) for the IDEAL IMPLANT is approximately linear with the measured diameter between 12.3 and 14.4 cm.

Another observation from this analysis is that the geometric model of Equation (4) is least accurate at low loads. This is seen in Figure 3 as the data points fall below the −2% line. In particular, the two data points for IDEAL IMPLANT and Allergan cohesive gel below the −2% line are at loads of 0.445 N, with errors of −4.8% and −7.1%, respectively.

### 3.3. Validation of Quasi-Equilibrium Assumption

The dynamic compression testing and associated analyses use the experimentally measured H values, as a function of load F, as an implant is continuously compressed to calculate D from Equation (4). To make the comparison between the static and dynamic compression testing, it was assumed that the dynamic compression process is a quasi-equilibrium process. A quasi-equilibrium process is one in which the deviation from equilibrium is infinitesimal, and all the states the implant passes through during the transient process may be considered equilibrium states. Therefore, this assumption constrains all implant properties to be constants for every instantaneous compression load during the dynamic process. This includes the implant diameter D, implant projection H, surface area A, and overall implant shape.

Another point of note is the manner in which the results are presented. Load-deformation data obtained from compression tests on different materials do not give a direct indication of material behavior because they depend on the initial material geometry. Hence, the data can be converted to compressive stress (i.e., load per area) and strain (i.e., normalized dimensional change) for presentation. For the present study, the three implants were chosen to essentially have the same initial geometry as summarized in Table 3. Therefore, it is appropriate to compare implant deformation directly as a function of the compressive load.

**Figure 4:**
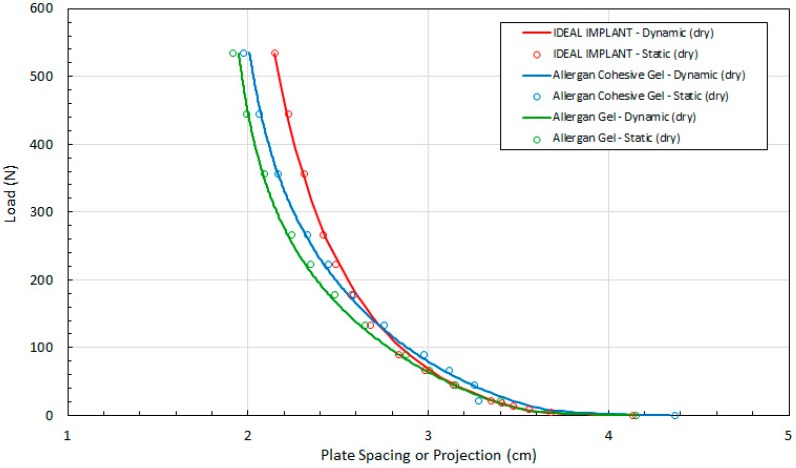
Load (F) as a Function of Plate Spacing (H) for Static and Dynamic Testing and Dry Platens.

The first step in the validation of the quasi-equilibrium assumption was to compare the implant projection (plate spacing) H recorded for the static and dynamic tests versus the compression load. The raw data for the static and dynamic tests for dry platens are presented in Figure 4 which shows the load F versus the plate spacing H for the three implants. The dynamic curves in Figure 4 were constructed from at least 48 experimental data points. The static data presented in Figure 4 were constructed from at least 12 experimental measurements. These data were then used to determine all of the other implant geometric properties, multi-dimensional strains, stresses and tangent moduli. The same implant was used for the static and dynamic tests, and as expected, there is close agreement between the static and dynamic data, indicating that the quasi-equilibrium assumption is valid. The distributions are very uniform and consistent, with average differences between the dynamic and static projection measurements at constant load F for the IDEAL IMPLANT, gel implant and cohesive gel implant of 0.59%, −0.52%, and 0.06%, respectively.

Referring to Figure 4, the measured implant projection H for the IDEAL IMPLANT and cohesive gel implants are similar up to about 100 N. Note, however, that the initial measured projection at 0.445 N load for the Allergan cohesive gel implant is about 5% higher than the IDEAL IMPLANT and Allergan gel implant (see Table 3), which should be taken into account when directly comparing the curves. 

**Figure 5:**
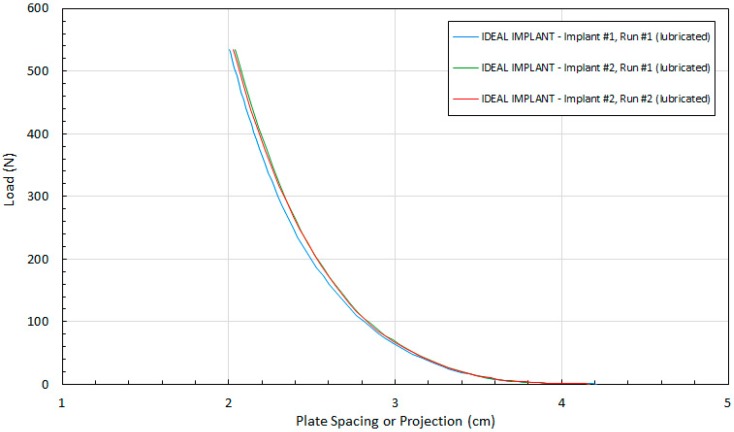
Reproducibility of Implant Projection Measurements.

The excellent reproducibility of the test method is demonstrated in Figure 5. Figure 5 shows the tight agreement between implant projection and load for two different IDEAL IMPLANTs and duplicate runs with the same IDEAL IMPLANT. Note that the surface of “Implant #1, Run #1” was not completely dry before commencing the test, so the lubrication for this IDEAL IMPLANT consisted of a combination of silicone oil and any residual moisture on the surface of the IDEAL IMPLANT. The relative standard deviation of the three runs from their mean is ±0.56%, highlighting the consistency of the measurement method between implants and for replicate runs with the same implant.

**Figure 6:**
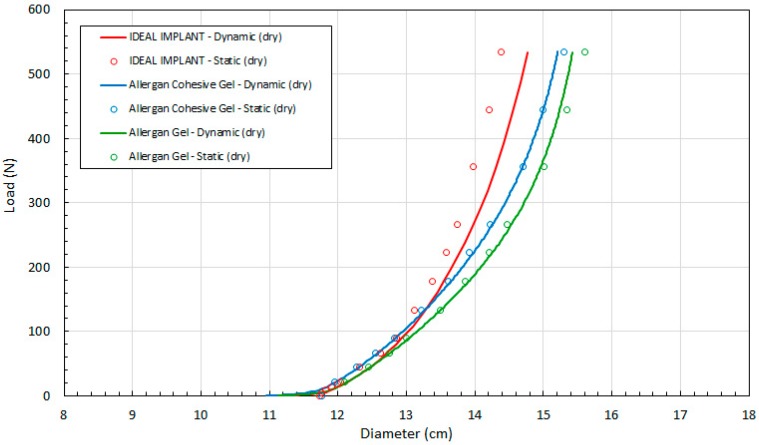
Load (F) as a Function of Implant Diameter (D) for Static and Dynamic Testing and Dry Platens.

To further validate the geometric model of Equation (4) for the implants, the experimental values of diameter D measured during the static tests were compared to the theoretical values of diameter D calculated using the dynamic compression values for H and Equation (4). The dynamic testing was conducted between loads of 0.445 N and 534 N. As shown in Figure 6, the agreement between the experimental and theoretical values of D is very good for all three implants over the entire load range from 0.445 N to 534 N. For the cohesive gel implant, the calculated values of D are within about 7% of the static measurements for D at 0.445 N and within about 0.6% for loads between 22.2 N and 534 N. For the gel implant, the calculated values of D are within about 1.1% or less of the static measurements for D for loads between 22.2 N and 534 N. For the IDEAL IMPLANT, the calculated values of D are within about 5% of the static measurement for D at 0.445 N and within about 2.5% for loads from 22.2 to 534 N.

Over the 22.2 N to 534 N range, the average error for calculated D values from the dynamic H measurements relative to the static measurements for D are 1.7%, 0.13% and −0.003% for the IDEAL IMPLANT, Allergan cohesive gel and Allergan gel implants, respectively. These errors can be compared to the average geometric model errors for the implant diameter, which are 1.7%, 0.17%, and −0.25% for the IDEAL IMPLANT, Allergan cohesive gel, and Allergan gel, respectively, over the same load range. Based on this comparison, most if not all of the error associated with the quasi-equilibrium analysis can be accounted for by the geometric model error. Consequently, the quasi-equilibrium assumption is assumed to be valid with little or no further contribution to error in implant diameter when determined using the dynamic measurements for H in Equation (4).

It is speculated that the geometric model may not be as good for the IDEAL IMPLANT due to the influence of the inner lumen and the baffles. Nonetheless, it can be concluded that the developed geometric implant model and quasi-equilibrium assumption for implants undergoing compressive loading produce very accurate results. The larger error at low loading might be expected since the implants do not conform as well to the flattened cylinder-outer torus geometry under very low compressive loads. As will be discussed later, the net effect of the slightly larger error in calculated D for the IDEAL IMPLANT dynamic measurements is to underestimate the shape stability of the IDEAL IMPLANT, making the shape stability differences of the IDEAL IMPLANT relative to the gel implants conservative.

It is interesting to note that the diameter D of the IDEAL IMPLANT at a given load is generally less than the diameters for the Allergan cohesive gel and Allergan gel implants, especially at loads greater than about 100 N. In particular, the IDEAL IMPLANT diameter is 2.8–7.8% and 0.7–6.1% smaller than the diameters of the gel implant and cohesive gel implant, respectively, over the load range of about 130 N to 534 N. In addition, the diameter of the cohesive gel implant is smaller than the diameter of the gel implant over the entire load range by 1.2–2.3%, indicating that the higher cross-link density associated with the cohesive gel has some effect on the geometry of the implant under compressive load. It is also noteworthy that the diameters for all implants were found to be similar up to about 100 N, suggesting that the IDEAL IMPLANT responds to loads similarly to gel implants at low compressive loads, even though it is filled with relatively inviscid saline fluid.

In summary, this new procedure for evaluating the geometric and mechanical properties of implants under compression is reproducible with a relative standard deviation less than 0.6%, and the accuracy of the theoretical model in predicting the diameter of an implant is considered to be within about ±2% for compression loads greater than 5 N. Both the geometric model for the implant as well as the quasi-equilibrium assumption were validated, and the quasi-equilibrium assumption was found to introduce little or no additional error outside the error introduced by the Equation (4) geometric model.

### 3.4. Dry Versus Lubricated Platens

**Figure 7:**
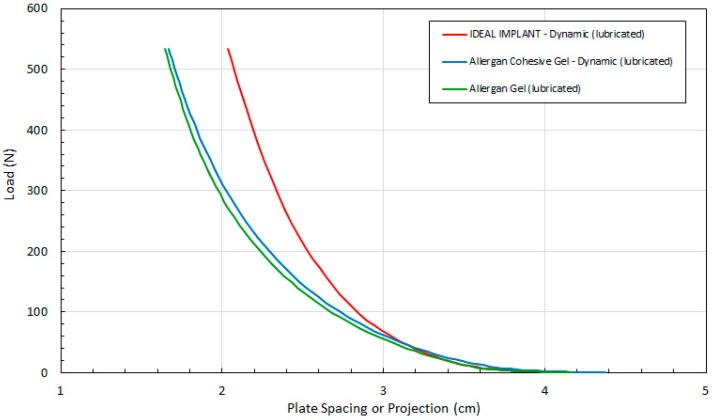
Load (F) as a Function of Plate Spacing (H) for Dynamic Testing and Lubricated Platens.

To evaluate the contribution of the frictional force between the platens and an implant, dynamic testing was also performed when both platens were lubricated with silicone oil (DPDM 400). As far as could be determined, no previous published breast implant study has considered the effect of lubricated platens on the response of implant shape due to compressive loading. The raw data for the dynamic tests using lubricated platens are presented in Figure 7 which shows the load versus the plate spacing for the three implants. Each curve was constructed from at least 52 experimental data points.

Again, these data are needed to determine all of the other implant geometric properties (i.e., D and A per Equations 4 and 5, respectively). The implant projection H for all three implants is similar up to about 50 N. Higher loads have a less pronounced effect on the projection of the IDEAL IMPLANT. Compression has a greater effect on the geometry of the two Allergan gel implants over the remaining load range. The distributions for the two Allergan gel implants are also similar over the entire load range, with the Allergan cohesive gel implant retaining a slightly higher projection at a given load. The minor differences in projection for the two Allergan gel implants suggests only a minor effect due to the higher gel cross-link density of the Allergan cohesive gel implant. As with the implant projection curves of Figure 4 for dry platens, note that the initial measured projection at 0.445 N load for the Allergan cohesive gel implant was about 5% higher than the IDEAL IMPLANT and Allergan gel implant (see Table 3), which should be taken into account when directly comparing the curves in Figure 7.

**Figure 8:**
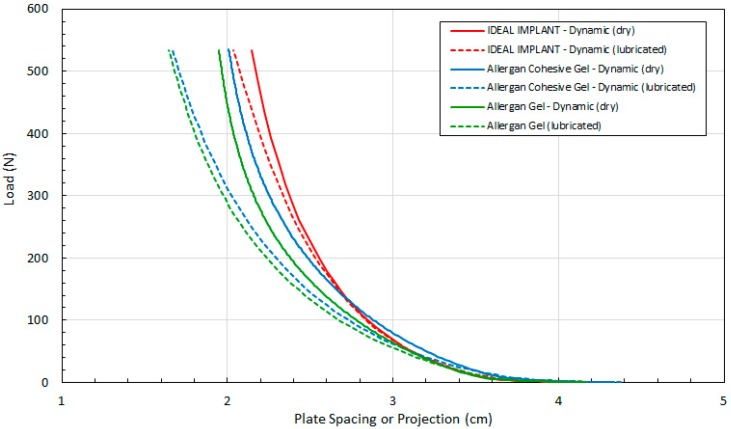
Load (F) as a Function of Plate Spacing (H) for Dynamic Testing, Dry and Lubricated Platens.

Figure 8 compares load F as a function of measured H for dry and lubricated platens and all 3 implants. The data in Figure 8 are arranged in pairs of solid and dashed lines for each implant, where the solid lines are the dry platen data and the dashed lines are lubricated platen data. For a given load, the implant projection for all three implants is smaller with lubricated platens, and this effect increases with increasing load. The differences in projection H for lubricated and dry platens are more pronounced for the Allergan cohesive gel and gel implants.

The implant projection at a given load F is also similar for all implants, and for lubricated and dry platens, for loads up to about 50 N. Again, although these similarities are noted, the Allergan cohesive gel implant had an initial measured projection at 0.445 N that was about 5% higher than the IDEAL IMPLANT and Allergan gel implant, which should be considered when directly comparing the curves of Figure 8.

**Figure 9:**
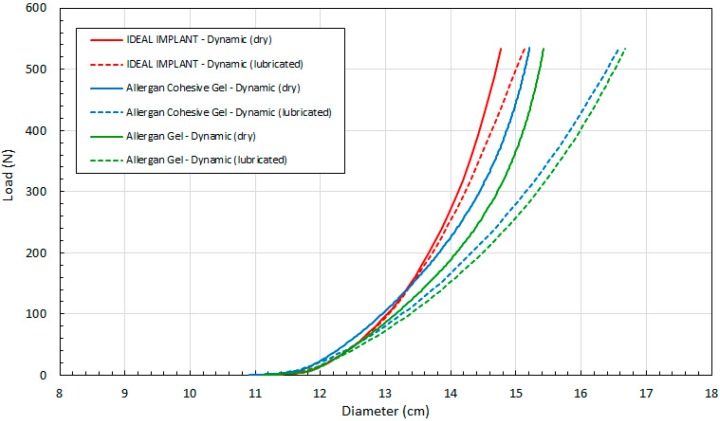
Load (F) as a Function of Implant Diameter (D) for Dynamic Testing, Dry and Lubricated Platens.

Figure 9 compares load F as a function of calculated D (Equation (4)) for all three implants. As in Figure 8, the data of Figure 9 are arranged in pairs of solid and dashed lines for each implant, where the solid lines are the dry platen data and the dashed lines are lubricated platen data. For a given load and implant, the diameter is larger when using lubricated platens. The difference between the dry and lubricated platen values of D increases with increasing load, and this difference is more pronounced for the two gel implants.

This data demonstrates that all three implants have similar diameters up to about 50 N, after which the IDEAL IMPLANT retains its diameter better than gel implants at a given load for both dry (solid lines) and lubricated (dashed lines) platen data. The differences between the dry and lubricated platen data for both gel implants are similar. As explained earlier and shown by Figure 3, the diameter of the IDEAL IMPLANT is actually overestimated by the theoretical calculation for D (Equation (4)), so the differences in diameters between the IDEAL IMPLANT and gel implants depicted in Figure 9 are likely conservative and even more pronounced.

**Figure 10:**
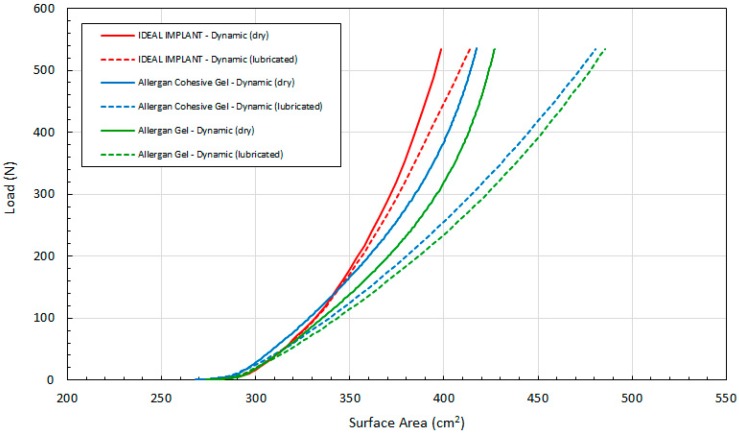
Load (F) as a Function of Implant Surface Area (A) for Dynamic Testing, Dry and Lubricated Platens.

Lastly, Figure 10 compares load F as a function of calculated surface area A per Equation (5) for all 3 implants. Lubricated platens result in a larger implant surface area for a given load, and the relative difference between the IDEAL IMPLANT and gel implants increases with the load. The two gel implant curves have a similar shape, but the calculated surface area of the gel implant is greater than the calculated surface area of the cohesive gel implant for both dry and lubricated platens. The surface area differences between the IDEAL IMPLANT and gel implants are very pronounced using lubricated platens, demonstrating that the gel implants deform considerably more than the IDEAL IMPLANT at a given load. Note also that per Equation (5), the area is a function of the calculated D and measured H. Since the calculated D per Equation (4) overestimates the statically measured D (see Figure 3, for example), the surface area A for the IDEAL IMPLANT shown in Figure 10 would also be overestimated. Consequently, the differences between the IDEAL IMPLANT and the Allergan gel implants are likely even more pronounced than that shown, since the surface area A is a quadratic function in D.

Since the external surface area of the implant, A, is what interacts with the breast capsule in vivo, this geometric property may have more physiological relevance to the interaction of the implant with the patient’s body. Although no research could be identified linking the implant surface area or implant surface area shape change to CC, the implant surface is the direct implant interface with the breast capsule wall. As shown in Figure 10, the external surface area of the IDEAL IMPLANT is generally less sensitive to load than the gel implants, meaning that the implant will move less (relative to the breast capsule wall) especially when subjected to loads greater than about 100 N. 

Several compression tests for FDA-approved breast implants have been conducted with dry platens in air. Overall, the results of this study show that the reduction in the frictional forces between the implant and surface imparting the load increases the implant shape change which will correspondingly reduce implant durability with respect to cyclic fatigue and ultimate strength. The exact implant surface friction conditions in vivo can only be speculated, but these results do demonstrate that frictional effects need to be appreciated and considered in laboratory implant testing.

### 3.5. Multi-Dimensional Strain and Tangent Moduli of Implants

The dynamic implant dimensional data were used to compute three types of implant strain: circumferential or diametric strain, projection strain, and surface areal strain. Engineering strain is the change in a dimension divided by the original dimension. Accordingly, three strains related to implant geometry are defined as a percentage change by:

(6) $$\mathrm{Projection}\;\mathrm{Strain}=\epsilon_{H}=-\frac{H-H_{o}}{H_{o}}\times 100\%$$

(7) $$\mathrm{Diametric}\;\mathrm{Strain}=\epsilon_{D}=\frac{D-D_{o}}{D_{o}}\times 100\%$$

(8) $$\mathrm{Areal}\;\mathrm{Strain}=\epsilon_{A}=\frac{A-A_{o}}{A_{o}}\times 100\%$$

In Equations (6)–(8), H_o_, D_o_ and A_o_ are the initial values for the plate spacing or implant projection, diameter and surface area, respectively, with a minimal initial load of 0.445 N. A negative sign is applied to projection strain since the implant projection decreases with load, and all strains are defined to be positive values. Each of the strains defined by Equations (6)–(8) yield a mathematical description of the implant shape response to compressive load, which in turn relates to the shape stability of an implant. Accordingly, the lower the strain value, the more shape stable the implant is to compressive loads.

**Figure 11:**
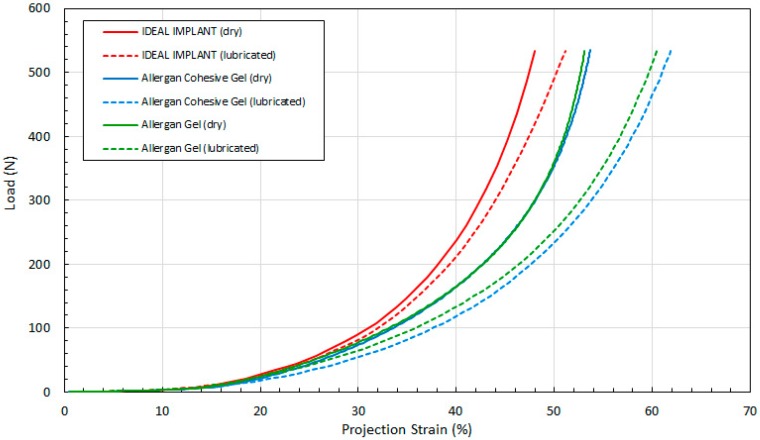
Load vs. Projection Strain, Dry and Lubricated Platens.

**Figure 12:**
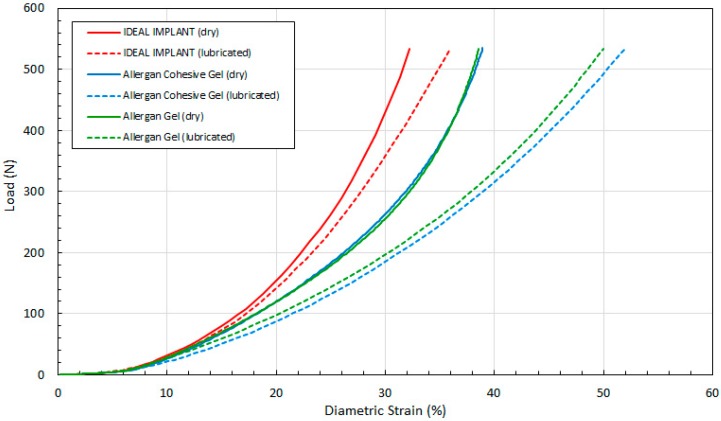
Load vs. Diametric Strain, Dry and Lubricated Platens.

**Figure 13:**
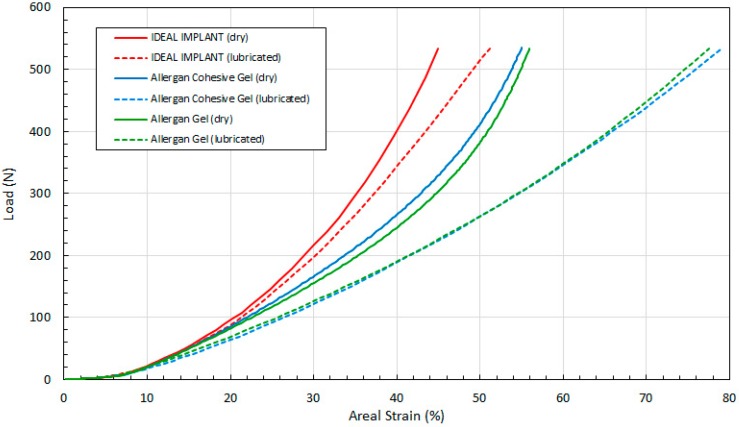
Load vs. Areal Strain, Dry and Lubricated Platens.

Figure 11 shows the load as a function of projection strain ϵ_H_ for all three implants, and for both dry and lubricated platens. Figure 12 shows the load as a function of diametric strain ϵ_D_ for all three implants, and for both dry and lubricated platens. Figure 13 shows the load as a function of areal strain ϵ_A_ for all three implants, and for both dry and lubricated platens. The data in the graphs are again presented in pairs, with the dry platen data as solid lines and the lubricated platen data as dashed lines.

Several observations are in order. First, all three engineering strains for the IDEAL IMPLANT are less than those for the Allergan cohesive gel and gel implants at a given load. Over the load range of about 100 N to 500 N, the projection strains ϵ_H_ of the gel implants relative to the IDEAL IMPLANT are about 10–15% and 13–22% higher for dry and lubricated platens, respectively. For the same load range, the diametric strains ϵ_D_ for the gel implants relative to the IDEAL IMPLANT are about 6–19% and 24–42% larger for dry and lubricated platens, respectively. And, for the same load range, the areal strains ϵ_A_ for the gel implants relative to the IDEAL IMPLANT are about 5–25% and 40–55% larger for dry and lubricated platens, respectively. Hence, the shape change due to compression is less for the IDEAL IMPLANT, especially for loads exceeding about 100 N. The trends in Figure 11, Figure 12 and Figure 13 are the same for both dry and lubricated platen data sets, except that the strain values are much larger for the case of lubricated platens. A consequence of these results is that the effect of platen surface friction could have an influence on cyclic and ultimate strength testing. Decreased surface friction would result in lower cyclic fatigue lifetime predictions and lower ultimate strengths.

Second, for the IDEAL IMPLANT, compression has the greatest effect on the projection and areal strain, with values ranging up to about 50% at 534 N for lubricated platens, and the least effect on the diametric strain, with values ranging up to about 35% at 534 N for lubricated platens. Third, the Allergan cohesive gel and the gel implants were found to have essentially the same shape response to compression, except the projection and diametric strains are slightly greater for the cohesive gel implant and lubricated platens, and the areal strain is somewhat larger for the gel implant and dry platens. The projection and diametric strains for dry platens and areal strain for lubricated platens are nearly identical for the two Allergan gel implants. The similar or nearly identical strains for the two Allergan gel implants was unexpected, since the cross-link density of the cohesive gel implant is higher than the gel implant.

An engineering compressive stress can be defined in terms of the initial implant planform area A_po_ = π D_o_^2^/4. This effectively normalizes the load by an initial implant size. The compressive stress S can therefore be defined in terms of the load F as:

(9) S=Compressive Stress=FApo

The planform areas for the IDEAL IMPLANT, Allergan cohesive gel, and Allergan gel implants are nearly identical at 108.0 cm^2^, 108.6 cm^2^ and 108.4 cm^2^, respectively. Consequently, a plot of this engineering compressive stress S for each of the engineering strains would show the same trends as in Figure 11, Figure 12 and Figure 13, except that the y-axis would be shifted down by these planform area factors. Note that this stress is not the stress experienced by the implant shell.

The slope of the stress-strain curves obtained from the data in Figure 11, Figure 12 and Figure 13 as a function of compressive stress can be used to calculate total implant tangent moduli. Engineering moduli are typically defined in terms of a ratio of stress to strain, and more typically taken as the slope of the initial linear portion of a stress-strain curve. For this application, however, we define these moduli as the local slope along the curve and not just the slope as the load approaches 0, thereby producing a tangent modulus that is a function of load F. In fact, the slope of the stress-strain curves for total implants is not linear, so this type of mathematical construct for the tangent moduli is appropriate. These tangent moduli, or slopes, are therefore defined as:

(10) Projection Tangent Modulus=ESH=ΔSΔϵH

(11) Diametric Tangent Modulus=ESD=ΔSΔϵD

(12) Areal Tangent Modulus=ESA=ΔSΔϵA

To compute these tangent moduli, the stress (S) from Equation (9) is first tabulated with the computed strains ϵ_H_, ϵ_D_, and ϵ_A_. Next, each successive pair of data sets for S and strain values are differenced and then ratioed as per Equations (10)–(12) to estimate the slope, or tangent, of the curve at the midpoint stress value for the data pair. These tangent moduli mathematically describe the shape stability of the total implant when subjected to a load, and have the units of pressure (e.g., kPa). The higher the modulus value, the more shape stable the implant is to compressive loads.

**Figure 14:**
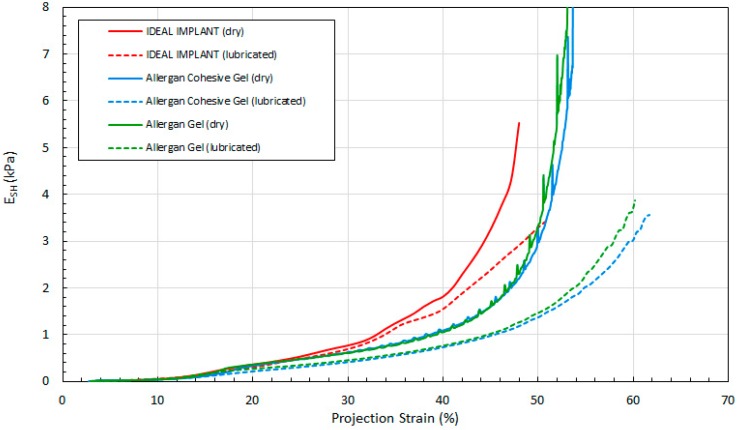
Projection Tangent Modulus with Projection Strain, Dry and Lubricated Platens.

**Figure 15:**
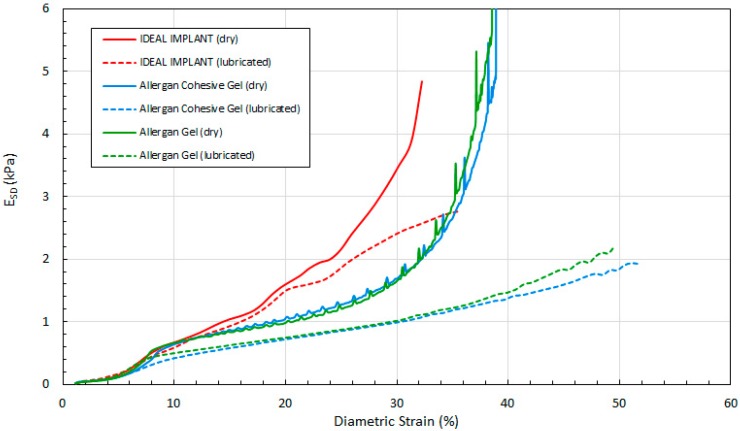
Diametric Tangent Modulus with Diametric Strain, Dry and Lubricated Platens.

**Figure 16:**
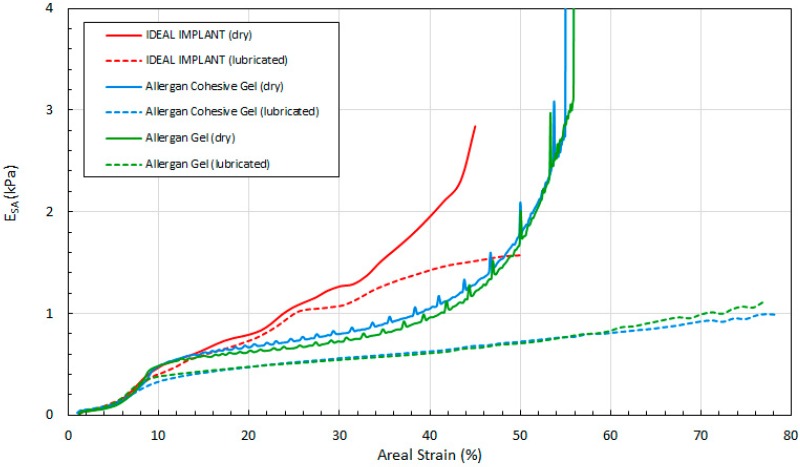
Areal Tangent Modulus with Areal Strain, Dry and Lubricated Platens.

Figure 14, Figure 15 and Figure 16 show the three tangent moduli as a function of respective strains for the three implants, dry and lubricated platens. The solid lines correspond to dry platen data and the dashed lines correspond to the lubricated platen data. Each curve was constructed from 45 to 207 data points calculated from experimental data using Equation (12). There is notably some noise in the Allergan gel and cohesive gel curves as a result of the differencing calculation of Equation (12), however the trends are clear and unaffected.

Several observations can be made regarding the tangent moduli trends of Figure 14, Figure 15 and Figure 16. First, at low strain and for both dry and lubricated platens, the tangent moduli for the IDEAL IMPLANT are similar to both gel implants. However, the projection, diametric and areal tangent moduli for the IDEAL IMPLANT diverge to higher values from the gel implants at about 25%, 12% and 14% strain, respectively, for dry platens, and at about 25%, 10% and 10% strain, respectively, for lubricated platens. This indicates that above these low strain values, the IDEAL IMPLANT is more shape stable than the cohesive gel and gel implants. For example, at 40% projection strain the IDEAL IMPLANT projection tangent modulus is about 80% and 100% higher than the gel implants for dry and lubricated platens, respectively. At 20% diametric strain, the IDEAL IMPLANT diametric tangent modulus is about 60% and 100% higher than the gel implants for dry and lubricated platens, respectively. And, at 40% areal strain, the IDEAL IMPLANT areal tangent modulus is about 80% and 130% higher than the gel implants for dry and lubricated platens, respectively. These differences in moduli increase even more at higher strain values, and are remarkable especially since the IDEAL IMPLANT is filled with an inviscid fluid rather than silicone gel.

A second observation is that the tangent moduli for the two Allergan gel implants are similar, and sometimes overlapping, over much of the strain range. There is, however, some divergence of the gel and cohesive gel curves at higher projection and diametric strain values, and between about 20% and 50% areal strain. The similarity in the gel implant tangent moduli suggests that despite the higher cross-link density of the cohesive gel, the two gel fillers in the gel implants result in similar shape stability.

For all three implants, another observation is that the tangent moduli for dry platens are higher than those for lubricated platens, although the effect is much more pronounced for the Allergan gel implants. This suggests that some of the resistance to deformation, creating larger values for the tangent moduli, is a result of the opposing frictional forces of dry platens. The lubricated platens reduce this force opposition to deformation thereby reducing these moduli for all implants. In essence, testing with dry platens may create an apparent shape stability in laboratory testing that is not entirely attributable to the implant itself.

### 3.6. Localized Strain at Very Low Compressive Loads (“Diametric Pinch”)

Desirable properties of a breast implant are to have a shape that that is minimally affected by compressive loading, while at the same time possessing a “soft” feel similar to a female breast. A responsive silicone gel implant is generally considered to be a standard biomaterial to mimic the softness of a female breast, so it would be beneficial to understand and quantify breast implant “softness”. Therefore, another objective of this study was to understand if implant shape stability has any correlation to implant softness.

In the foregoing compression analysis of breast implants, the focus was on total implant shape change resulting from compression loading. Implant softness is an implant property that may not be fully quantified by the foregoing total implant compression testing, especially since softness relates more to the resistance of an implant to localized pinching or squeezing at the perimeter across the implant diameter. The term localized is used in this context since a pinch or squeeze may involve a more concentrated diametrically opposed compression load on an implant, for example, and not a compressive load applied over a large portion of an implant as in platen compression. Accordingly, to complement the total implant compression testing, to understand the implant’s mechanical properties at very low loads, and to determine if there is any correlation between implant shape stability and implant softness, a test was developed to measure the force to pinch or squeeze implants by diametrically opposed silicone probes simulating fingertips. This testing provides further information about how an implant responds to unconfined, localized compressive forces when the implant can still retain some of its original geometry. This free deformation response to a compressive force therefore simulates the tactile, palpable, or softness properties of an implant, rather than the total implant mechanical properties measured in the foregoing analysis.

**Figure 17:**
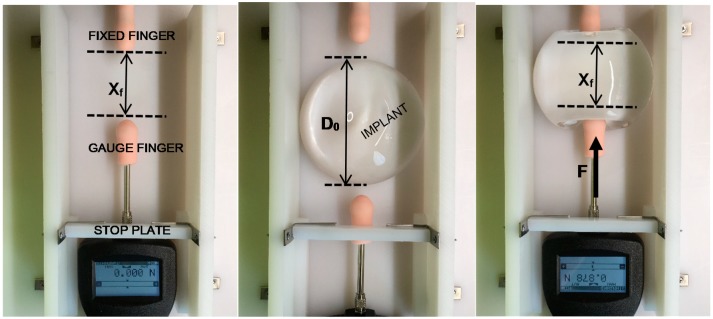
Setup for X_f_ (**left**), Implant Placement (**center**), Implant Pinched for Force Measurement (**right**).

Figure 17 shows the general setup for this testing. The center of the two probes is approximately 17 mm from the horizontal surface, and the diameter of the silicone probes are about 18 mm to simulate fingertips. The ultra-high molecular weight polyethylene (UHMWPE) surface is wetted so that the implant freely slides along it between the two silicone fingers. UHMWPE has a surface energy similar to polytetrafluoroethylene, so an implant can freely slide on this surface when wetted.

Pre-defined “stops” controlled by an adjustable stop plate limit the final position of the force gauge with attached finger, thereby defining a repeatable final squeeze or pinch distance, or distance between the fingers X_f_. The movable gauge and attached gauge finger are retracted and an implant is placed between the two silicone fingers. The movable gauge finger is then pushed into the implant and the implant slides into the fixed finger. By this simple test, the implant projection is not confined, so when the implant is diametrically squeezed or pinched, it is free to deform naturally in all directions in response to the localized compressive load. Figure 17 also shows a pinched implant using this method. The pinch force F is recorded as a function of final pinch distance, X_f_, and the test is repeated a total of 30 times for each implant and each X_f_. Curves were then generated for the three implants for the series of X_f_ values. A localized diametric strain, ϵ_LD_, can be defined from the initial implant diameter D_o_ and the final pinch distance X_f_ as:

(13) $$\mathrm{Localized}\;\mathrm{Diametric}\;\mathrm{Strain}=\epsilon_{\mathrm{LD}}=-\frac{X_{f}-D_{o}}{D_{o}}\times 100\%$$

The negative sign is used in Equation (13) because the pinch distance X_f_ is smaller than the initial implant diameter.

**Figure 18:**
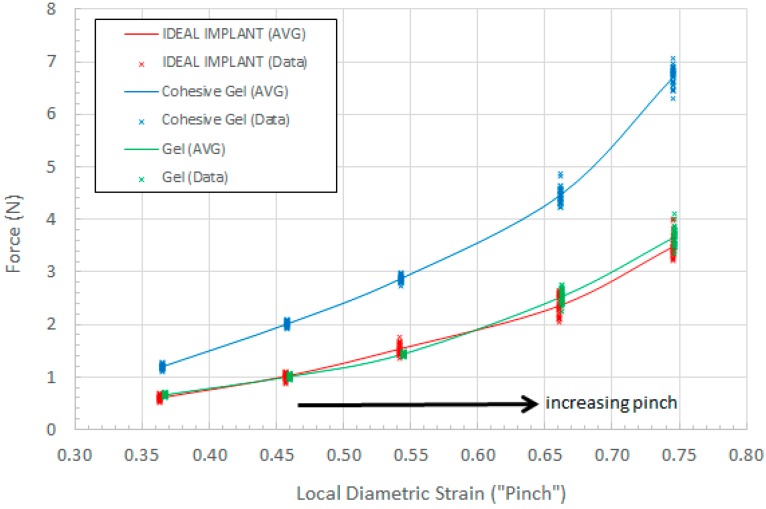
Pinch Force as a Function of Local Diametric Strain.

A total of 30 force measurements were made for each implant at each pinch distance X_f_. Five finger-to-finger spacings of 75 mm, 64 mm, 54 mm, 40 mm and 30 mm were tested, corresponding to ϵ_LD_ values of 0.35, 0.46, 0.54, 0.66 and 0.75, respectively. The force measurements were consistent with a standard deviation ranging from 2.0–8.0% relative to the mean for each 30-point data set. Figure 18 plots the force data as a function of the localized diametric strain. All data points (30) at each localized strain for each implant are shown, along with the curve drawn through the average force for each of these data sets.

As shown in Figure 18, for a given local diametric strain ϵ_LD_, the pinch force for the IDEAL IMPLANT is similar to the pinch force for the Allergan gel implant, which is considered to be a standard for softness comparison. The pinch force for the IDEAL IMPLANT is also found to be much lower than the Allergan cohesive gel implant pinch force. The measured force for each local diametric strain therefore provides a mathematical description of the implant tactility or palpability. For a given local diametric strain such as squeezing or pinching, a more compliant, or softer implant, would require a lower force to squeeze or pinch the implant.

A t-test (α = 0.05) was performed on the data sets to determine the statistical significance of measured force differences between the IDEAL IMPLANT and the Allergan gel implants. Statistically significant differences between the IDEAL IMPLANT and Allergan cohesive gel implant were found at each strain ϵ_LD_, with the mean pinch force for the Allergan cohesive gel being 88–98% higher than the IDEAL IMPLANT. There were also statistically significant differences between the IDEAL IMPLANT and Allergan gel implant pinch forces for all pinch distances except 64 mm (ϵ_LD_ = 0.54). The statistically significant pinch force differences between the Allergan gel implant relative to IDEAL IMPLANT ranged from −6% (less than the IDEAL IMPLANT) to 10% (greater than the IDEAL IMPLANT). Further transient testing for local diametric strain will be presented in a future publication, however this simple preliminary testing clearly demonstrates the relative trends related to tactility, palpability and softness between the IDEAL IMPLANT, Allergan gel and Allergan cohesive gel implants.

## 4. Discussion

This study has demonstrated a new methodology to assess and understand the multi-dimensional mechanical properties of total breast implants subjected to compressive loads. The technique and results may be used to assess how an entire implant shape responds geometrically to loads, and to compare the potential shape stability of implants. The shape stability of an implant can be mathematically described in terms of implant strains and tangent moduli which can be directly calculated using compressive force-displacement data.

The methodology used to determine the geometry of implants during compression was found to be both accurate and reproducible for the conditions considered in this study. From the experimentally measured plate spacing and compressive force data, and assuming a cylinder-outer torus composite geometry, implant diameters can be calculated to within about ±2% of the empirical diameters for loads greater than about 5 N. The implant diameter and plate spacing can then be used to accurately calculate the implant surface area as a function of load. This new method is also reproducible with a relative standard deviation of about 0.6%.

Aside from using this new methodology to compare the total implant mechanical properties of implants, it may also prove useful in more quickly evaluating the durability and lifetime impact of an implant design change (e.g., different profile or different fill percentage). For example, if a design change results in similar or lower multi-dimensional strains and/or similar or greater tangent moduli, the durability and lifetime of the implant as assessed through lengthier fatigue testing is likely to be unaffected or possibly improved.

Having demonstrated the accuracy and reproducibility of this method, implants of similar size representing the product lines of IDEAL IMPLANT, Allergan Natrelle INSPIRA gel and Allergan Natrelle INSPIRA cohesive gel were selected and tested. Implants manufactured today are of high quality with tight specifications for shell thickness and weight, so the tested implants were assumed to be representative of these commercial product lines. The test data was then analyzed using the geometric model presented in this study to further calculate strains and tangent moduli for the implants. Implants were tested using both dry and lubricated platens.

Multi-dimensional strains calculated using this methodology provide a means to assess the shape stability of the implants in response to compressive loads. Tangent moduli, which are essentially a local slope, or tangent, of a stress-strain curve, were also defined as an additional mathematical surrogate for shape stability. These tangent moduli reinforce the shape stability assessment provided by the multi-dimensional implant strains.

Over the load range of 100 N to 500 N, the projection strain ϵ_H_ for the Allergan gel implants relative to the IDEAL IMPLANT is about 10–15% and 13–22% larger for dry and lubricated platens, respectively. Over the same load range, the diametric strain ϵ_D_ for the Allergan gel implants relative to the IDEAL IMPLANT is about 6–19% and 24–42% larger for dry and lubricated platens, respectively. And, for the same load range, the areal strain ϵ_A_ for the Allergan gel implants relative to the IDEAL IMPLANT is about 5–25% and 40–55% larger for dry and lubricated platens, respectively. Hence, the implant shape change resulting from compressive loads is less for the IDEAL IMPLANT compared to the Allergan gel implants.

Tangent moduli were also calculated from the platen spacing data and geometric models for the implants during compression. The computed tangent moduli corroborated the trends found for the strains regarding the shape stability of the implants. Above strains of 10–25%, and depending on the particular tangent modulus, the tangent moduli for the IDEAL IMPLANT diverge to higher values compared to the Allergan gel implants. This means that as the implant dimensionally deforms more than 10–25%, the IDEAL IMPLANT becomes more resistant to further shape change compared to the gel implants. It is surmised that this divergent behavior is related to the dual-lumen structure of the implant, but further research is needed to deconvolute the mechanical properties of the inner and outer lumens when subjected to compressive loads.

The strains calculated for the implants provide potential insight into the unique clinical results for the IDEAL IMPLANT. Both the diametric and projection strains affect the maximum stress in the perimeter of the outer implant shell. The greater these values are, the greater the maximum shell stress. Hence, the IDEAL IMPLANT would be expected to have the lowest value of maximum shell stress for a given load. (Note that this perimeter shell stress is not to be confused with the compressive stress S defined by Equation (9).) This means that the IDEAL IMPLANT is more durable under compressive cyclic loads and ultimate strength compressive loads (i.e., ultimate strength) since failure occurs at the implant perimeter during compression loading. This increased durability would be reflected in a longer cyclic fatigue lifetime and a greater ultimate strength for the IDEAL IMPLANT compared to the two gel implants, assuming all three implants have comparable tensile strength and shell thickness. Further, if this perimeter stress somewhat correlates to rupture rates, then this could help explain the lower rupture rates observed clinically for the IDEAL IMPLANT.

The ultimate strengths of the IDEAL IMPLANT have also been measured to be very high (i.e., exceeding 15.2 kN), and believed to be higher than the ultimate strength of all commercially available breast implants. This property of the IDEAL IMPLANT parallels the higher tangent moduli determined from this study. The lower multi-dimensional strains and higher tangent moduli of the IDEAL IMPLANT under compressive load may also correlate to a higher resistance for implant folding or flexing, which could lead to fewer or no fold flaw failures as observed clinically.

The multi-dimensional strains and tangent moduli also relate to potential movement or shape change of the implant in vivo when subjected to compressive loads. In particular, lower multi-dimensional strains and higher tangent moduli, as with the IDEAL IMPLANT, implies that an implant will move or change shape less within the capsule and against the capsule wall. If a reduction in shape change with repeated compressive loads corresponds to a reduction in repeated dynamic interaction between the implant surface and the capsule wall leading to a reduction in repeated micro-trauma to the capsule wall, then this might explain at least in part why the IDEAL IMPLANT exhibits lower CC rates.

The multi-dimensional strains and tangent moduli of the Allergan gel and Allergan cohesive gel implants were also found to be similar. For example, the projection and diametric strain curves overlap for dry platens, and the areal strain of the two gel implants overlap for lubricated platens. Although there were some differences between the gel and cohesive gel implants for projection and diametric strain using lubricated platens and with areal strain using dry platens, these differences were not as great as those differences between the two gel implants and the IDEAL IMPLANT. Similar trends and overlap can also be seen in the tangent moduli for the gel implants. The similar multi-dimensional strains and tangent moduli for the Allergan gel and Allergan cohesive gel implants seem to suggest that the degree of gel filler cross-linking may have limited impact on shape stability under compressive loads. This result was unexpected, and contrary to conventional thought regarding cohesive gel implants.

The results of this study demonstrate that the overall breast implant multi-dimensional strains are important mechanical properties that not only influence the shape response due to compression, but also affect the shell stress and the subsequent cyclic fatigue lifetime, and the implant ultimate strength. Perhaps the multi-dimensional strains and tangent moduli should be adapted into standardized testing for the comparisons and evaluation of breast implant mechanical properties. These properties could help improve the understanding of the shape deformation characteristics of breast implants.

The frictional interaction of implants with platens during compression testing was shown to have an effect on total implant mechanical properties and implant geometry. The in vivo environment surrounding an implant is expected to be somewhat lubricious, however, the exact nature of this lubriciousness and resulting frictional interaction with an implant can only be speculated. Overall, it is concluded from this study that a lubricious interface between an implant and the compressive load interfacing surface has an appreciable effect on the resulting deformation mechanics and geometry of an implant.

Lastly, a simple “pinch” test was developed to describe the tactile differences between implants at very low (i.e., <8 N) and localized compressive loads. The pinch force for the Allergan gel and IDEAL IMPLANT were observed to be very similar, and much lower than the Allergan cohesive gel implant. The localized diametric strain is a metric which describes implant deformation resulting from localized compressive forces across the implant diameter applied at the perimeter, and was developed in this study as a potential correlative to implant “feel” or softness. The results from this test provide more objective evidence that the IDEAL IMPLANT^®^ does indeed feel like a gel implant, supporting what has long been claimed by plastic surgeons and patients who compared the IDEAL IMPLANT side-by-side with gel implants. Despite the higher shape stability of the IDEAL IMPLANT, this testing showed that the IDEAL IMPLANT still has the feel of a gel implant and is more compliant than a cohesive gel implant.

## 5. Conclusions

A new methodology was developed and demonstrated that can provide a means to evaluate the mechanical properties of implants. This new methodology was found to be both accurate and reproducible, requiring only compressive load and platen spacing data which can be continuously monitored and recorded digitally during an automated test. This method was successfully used to evaluate and compare the IDEAL IMPLANT to silicone gel implants having a similar size.

This study provides an additional tool with which breast implants can be characterized, beyond the realm of traditional testing. The methods introduced and validated in this study may also provide physical and mechanical properties related to breast implant durability and safety. Future testing using this methodology may help in understanding the linkage between implant mechanical properties and clinical outcomes. This methodology may also provide the means to assess and optimize current and future implant designs toward improved clinical outcomes.

Compared to gel implants of similar size, the IDEAL IMPLANT has lower multi-dimensional strains and higher tangent moduli especially for loads exceeding about 100 N. The shape stability of the IDEAL IMPLANT described mathematically by these factors may help explain the higher ultimate strength of the IDEAL IMPLANT, the absence of clinical fold flaw failures, and the favorable clinical outcomes of lower CC rates and lower deflation/rupture rates, especially those related to shell failures. Despite the higher shape stability of the IDEAL IMPLANT relative to gel implants, at low, localized compressive loads the IDEAL IMPLANT exhibits palpability similar to a gel implant, and is much more compliant to localized compressive forces than the cohesive gel implant.

The total implant compression testing and the pinch testing of this study provide an additional level of understanding of breast implant mechanical properties not currently accounted for by fatigue testing and materials testing per the relevant ASTM and ISO standards. This study shows that implant softness does not necessarily correlate to low shape stability, and that shape stability is not a property held exclusively by gel implants. The shape stability of an implant dictates how an implant will retain its shape, move and deform within and against the breast capsule when subjected to compressive loads. Further research is needed to understand any potential links between shape stability and CC, however, a higher shape stability should have direct and positive implications for the ultimate strength and durability of an implant under compressive loads. Higher shape stability may even play a role in reduced failures such as fold flaws, since a higher shape stability implant would have less tendency to wrinkle. The pinch testing also demonstrated that an implant such as the IDEAL IMPLANT, which was found to have a higher shape stability than the gel implants, can still feel as soft as a gel implant.

## Figures and Tables

**Table 1:** Deflation/Rupture (MRI Cohort). FDA trial data for primary augmentation.

Breast Implant	Time Frame	Kaplan-Meier Risk Rate of 1^st^ Occurrence
IDEAL IMPLANT	8 year	2.1%
Allergan Gel	8 year	7.4%
Mentor Gel	8 year	13.6%
Sientra Gel	8 year	7.2%

**Table 2:** Capsular contracture Baker III/IV. FDA trial data for primary augmentation.

Breast Implant	Time Frame	Kaplan-Meier Risk Rate of 1^st^ Occurrence
IDEAL IMPLANT	8 year	6.6%
Allergan Gel	7 year	16.2%
Mentor Gel	8 year	10.9%
Sientra Gel	8 year	11.2%

**Table 3:** Tested implants and their properties.

Implant	Size ^a^	Type	D_o_ (cm)	H_o_ (cm)	V (cm^3^)	t (mm) ^b^
IDEAL IMPLANT^®^ Structured Breast Implant	335 cc	Smooth, round	11.73	4.14	346	0.386
Allergan Natrelle INSPIRA^®^ Gel	345 cc	Style SRF, smooth, round, full	11.76	4.36	345	0.419
Allergan Natrelle INSPIRA^®^ Cohesive Gel	345 cc	Style SCF, smooth, round, full	11.75 ^c^	4.16	345	0.462

(a) Implant size as provided on shelf box label. (b) Shell thickness t is an average of 4 equally spaced anterior perimeter measurements on implants after testing. (c) Diameter on Allergan shelf box label.

**Table 4:** Typical loads on a woman’s breast & breast implants.

Activity	Load on Breast or Implant (N)	Source
Standing	1.3–8.3	Gefen (2007)
Supine	4.9–9.8	Gefen (2007)
Kneeling (on floor)	4.9–9.8	Gefen (2007)
Walking	2.3–15	Gefen (2007)
Running: stair climbing	7.8–50	Gefen (2007)
Vertical jumping—free	3.9–25	Gefen (2007)
Vertical jumping—trampoline	9.1–58	Gefen (2007)
Lying face down, embracing, hugging	156 N	Assume 1/4 of a 623 N body weight imposed on implant(s). Mentor Corporation [25] suggests ¼ body weight.
Mammography procedure	49–187 N	Sullivan (1991)
CPR procedure	445–556 N	Geddes (2007)

## Abbreviation

A	Exterior surface area of implant
A_o_	Initial external surface area under 0.445 N load on flat surface
A_po_	Initial planform area of implant
ALCL	Anaplastic Large Cell Lymphoma
BIA	Breast implant-associated
BII	Breast implant illness
BTC	Biomechanical tissue characterization
CC	Capsular contracture
d	Diameter of platen contact with implant anterior and posterior surface
D	Implant diameter during testing
D_o_	Initial diameter of implant under 0.445 N load on flat surface
ϵ_A_	Areal strain
ϵ_D_	Diametric strain
ϵ_H_	Projection strain
ϵ_LD_	Localized diametric strain
E_SA_	Areal tangent modulus
E_SD_	Diametric tangent modulus
E_SH_	Projection tangent modulus
F	Applied load
H	Plate spacing or implant projection during compression testing
H_o_	Initial projection, or height, of implant under 0.445 N load on flat surface
PMA	Pre-Market Approval
S	Compressive stress
t	Average shell thickness at perimeter
V	Implant volume including fill and shells
X_f_	Finger-to-finger diametric distance
